# Inhibiting the “isolated island” effect in simulated bone defect repair using a hollow structural scaffold design

**DOI:** 10.3389/fbioe.2024.1362913

**Published:** 2024-04-02

**Authors:** Xiao Liu, Jianpeng Gao, Jianheng Liu, Licheng Zhang, Ming Li

**Affiliations:** ^1^ Department of Orthopaedics, The Fourth Medical Center of the Chinese PLA General Hospital, Beijing, China; ^2^ National Clinical Research Center for Orthopedics, Sports Medicine and Rehabilitation, Beijing, China

**Keywords:** bone defect repair, bone tissue engineering, autografts, osteogenesis, hollow structure

## Abstract

The treatment of bone tissue defects remains a complicated clinical challenge. Recently, the bone tissue engineering (BTE) technology has become an important therapeutic approach for bone defect repair. Researchers have improved the scaffolds, cells, and bioactive factors used in BTE through various existing bone repair material preparation strategies. However, due to insufficient vascularization, inadequate degradation, and fibrous wrapping, most BTE scaffolds impede new bone ingrowth and the reconstruction of grid-like connections in the middle and late stages of bone repair. These non-degradable scaffolds become isolated and disordered like independent “isolated islands”, which leads to the failure of osteogenesis. Consequently, we hypothesized that the “island effect” prevents successful bone repair. Accordingly, we proposed a new concept of scaffold modification—osteogenesis requires a bone temporary shelter (also referred to as the empty shell osteogenesis concept). Based on this concept, we consider that designing hollow structural scaffolds is the key to mitigating the “isolated island” effect and enabling optimal bone regeneration and reconstruction.

## 1 Introduction

Annually, millions of people worldwide experience bone tissue defects owing to trauma, bone disease, tumor resection, osteoporosis-related fractures, congenital bone deformities, and aging (Chen et al., 2018). The treatment of bone tissue defects remains a complicated clinical challenge (Kobbe et al., 2020). Autologous bone grafts are considered the gold standard for treating bone defects (Zhu et al., 2021), and remain the most efficacious approach for bone regeneration (García-Gareta et al., 2015). Currently, the anterior iliac crest (AIC) is the most common source for obtaining autologous bone for grafting (Ahlmann et al., 2002). The obtained autologous bone is segmented into smaller bone chips and subsequently inserted into the site of irregular bone defects, where it serves as a scaffold, filling the gaps and facilitating bone healing *via* osteoconductive (Stanovici et al., 2016). However, the use of autografts poses various challenges, including a lack of bone supply, protracted surgical duration, and complications arising at donor sites (neurovascular damage, hematoma, infection, cosmetic disfigurement, and post-operative discomfort). Collectively, these factors hinder autograft utilization (Almubarak et al., 2016; Liu and Lv, 2018; Cunha et al., 2021).

Owing to breakthroughs in bone tissue engineering (BTE), researchers have gained an in-depth understanding of bone physiology and the mechanisms underlying bone healing (Zhu et al., 2021). Various biomimicking and bioinspired BTE scaffolds have been developed to overcome autograft-related challenges and provide new strategies for bone defect repair and reconstruction (Liu et al., 2011). However, BTE scaffolds are associated with several limitations, such as insufficient vascularization in the center of the BTE scaffold (Valtanen et al., 2021), limited ingrowth of new bone (He and Ye, 2012), inadequate degradation (Li et al., 2019; Pepelassi et al., 2019), and scaffold fiber wrapping (He et al., 2020), which all negatively influence osteogenesis. Extensive efforts have been made to enhance the three essential components of BTE (cells, scaffolds, and bioactive factors) using various modification strategies (Du et al., 2019). These strategies aim to optimize scaffold shape, size, pore size, porosity, composition ratio, bioactive factor loading, and inclusion of stem cells. Despite these endeavors, existing scaffolds still do not meet the desired expectations. Hence, the large majority of BTE scaffold research remains confined to laboratory settings and the scaffolds have not been clinically applied. Therefore, we aim to develop strategies that enable BTE scaffolds to attain bone reconstruction efficacy comparable to that of autografts. We sought to identify factors that impede bone regeneration within BTE scaffolds. Furthermore, we aimed to identify a novel and innovative design concept that can overcome the current limitations in BTE scaffold design.

In clinical practice, we have discovered that bone reconstruction is effective when AIC bone chips are used for bone filling. It was accidently discovered, through X-ray and CT observations, that the autogenous bone chips were initially implanted in a dispersed and disordered fashion, resembling independent “isolated islands”. Subsequently, the newly formed bone interconnected with the “isolated islands” to form a grid-like network, ultimately culminating in optimal bone reconstruction. In contrast, owing to the non-autologous nature of bionic BTE scaffolds, we observed that the growth of new bone was primarily limited to the surface or inward growth upon implantation. Over time, the BTE scaffolds lose their initial predetermined optimal structure as a consequence of disordered degradation. The remaining scaffolding inevitably hinders the ingrowth of new bone and the reestablishment of a three-dimensional grid-like network. This defect is predominantly attributed to the absence of vascularization within the scaffold and encapsulation of inflammatory fibers, as mentioned previously. The residual scaffold can be metaphorically compared to an “isolated island”; thus, leading to a negative impact on bone regeneration during the intermediate and advanced phases.

## 2 Hypothesis

We hypothesized that the “isolated island” effect underlies the prevention of bone regeneration and reconstruction when using BTE scaffolds. Hence, we proposed an innovative modification method wherein osteogenesis is based on a temporary shelter, or the empty shell osteogenesis concept. According to this concept, optimal BTE scaffolds should offer specific mechanical reinforcement during the initial phase of bone healing, imitate autogenous bone chips to guide bone regeneration, establish a temporary osteogenic platform, and stimulate inherent osteogenic potential during the intermediate and late stages of the bone reconstruction process, thus achieving bone repair comparable to that with autografts.

## 3 Discussion

Current material design concepts can be improved according to the “isolated island” hypothesis. The current research strategy stems from a materials science approach which focuses on the improvement and modification of the three critical BTE elements (cells, scaffolds, and bioactive factors). Currently, most traditional BTE scaffold modification strategies are restricted to adjusting the parameters of the material itself, such as pore size and porosity, surface morphology, and composition ratio (Turnbull et al., 2018). However, BTE scaffold utilization is still associated with challenges; for example, vascular endothelial and mesenchymal cells cannot effectively grow into the scaffolds (Wang et al., 2019; Yin et al., 2019; Valtanen et al., 2021), and fibrous encapsulation can occur following the immune response (Freytes et al., 2013). Consequently, this may result in the formation of “isolated islands”, which, unlike autologous bone chips, hinder the effective formation of new bone. Moreover, aligning the rate of material degradation with the rate of bone formation remains challenging (Duda et al., 2023). Thus, the design concept needs to be revolutionized for clinical application. The material should be designed and developed from the perspective of the pathophysiological process of bone repair.

In autologous bone transplantation, AIC-harvested bone blocks are cut into small granular pieces and used to fill the bone defect area. When they are macroscopically observed, the cut pieces become independent bone pieces that connect with others to provide mechanical and structural support to the area of bone regeneration. At a microscopic level, osteogenesis-related cells and extracellular matrix penetrate the bone fragments. The fragments provide support for cell adhesion, proliferation, and differentiation, as well as favorable conditions for angiogenesis and nutrient transport (Burchardt, 1983; Weber, 2019). Finally, new bone is gradually formed through creeping substitution (Weber, 2019). Based on these findings, we postulate that the design of BTE scaffolds must incorporate three key considerations. Firstly, BTE scaffolds should emulate autologous bone fragments, which primarily serve as a transient platform for cells involved in bone regeneration (Lopes et al., 2018; Wang et al., 2020). This platform should be conducive to blood vessel invasion, cell migration, proliferation, differentiation, and communication (Zhu et al., 2021), and provide a favorable extracellular matrix environment for subsequent new bone formation (Langer and Vacanti, 1993; Khan et al., 2012). Secondly, it is imperative that BTE scaffolds effectively retain and stimulate the inherent regenerative capacity of bone tissue. This is primarily attributed to the robust reparative and regenerative potentials of bone tissue following pathological injury (Qiao et al., 2021). Specifically, the activation of cellular cascades within the affected bone tissue region plays a crucial role in facilitating systematic healing, ultimately leading to reconstruction of bone structure and restoration of its functionality (Taichman, 2005; Sordi et al., 2021). Third, the BTE scaffolds should offer adequate space for the growth of new bone and infiltration of neovascularization, creating an optimal space for the ingrowth of new bone tissue and formation of a highly interconnected vascular network.

**FIGURE 1:**
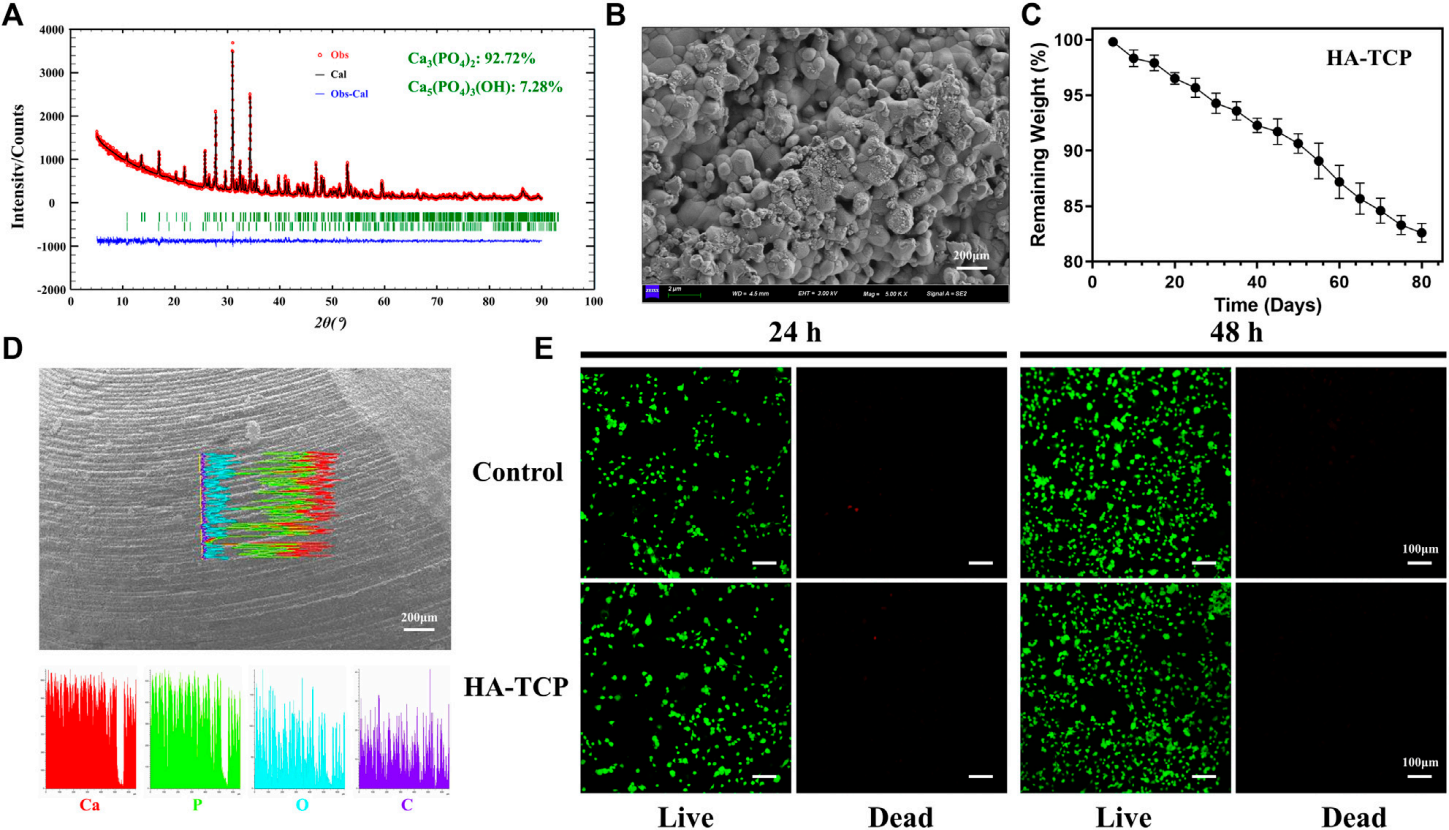
Characterization and biocompatibility analysis. **(A)** X-ray diffraction (XRD) analysis revealed that the scaffold primarily comprises tricalcium phosphate (Ca_3_(PO_4_)_2_, 92.72%) and hydroxyapatite components (Ca_5_(PO_4_)_3_(OH), 7.28%). **(B)** Scanning electron microscopy (SEM) observation of scaffold surface characterized by rough morphology. **(C)** Testing the biodegradation rate of the scaffold in simulated body fluids. **(D)** The elemental composition of the scaffolds was analyzed using energy dispersive spectroscopy (EDS). The results obtained from the line scans revealed that the scaffold surfaces primarily comprised Ca and P elements. **(E)** The live/dead staining analysis of MC3T3-E1 cultured in scaffold extracts for 24 and 48 h revealed a significant increase in live cells compared to dead cells. Additionally, cell count was significantly increased at 48 h compared with that at 24 h. These findings suggest that the scaffolds did not exhibit noticeable cytotoxic effects.

**FIGURE 2:**
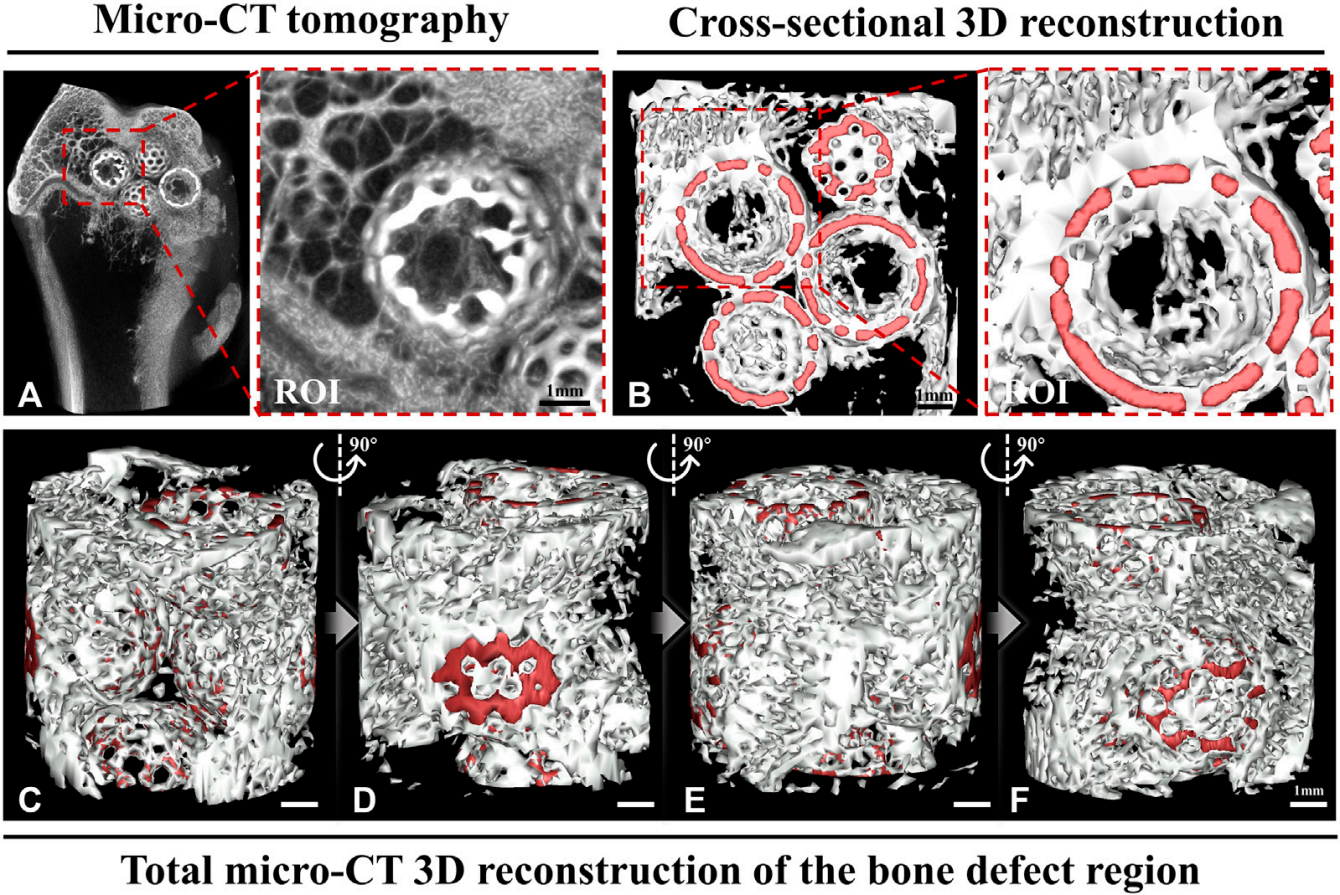
Micro-computed tomography (CT) and a three-dimensionally (3D) reconstructed image. **(A)** The micro-CT image shows that the hollow structural scaffolds serve as a temporary osteogenic platform, facilitating the growth of a grid-like bone network on their surface and within their interior. The region of interest (ROI) exhibits the growth of a grid-like trabecular reconstruction structure within the hollow scaffold, resembling the normal structure found externally. **(B)** The cross-sectional 3D reconstruction shows the formation of grid-like neoplastic bone structures within and outside the hollow structural scaffolds in the area of bone defects. The ROI demonstrates the interconnectedness of the neoplastic bone within the spherical scaffold in a 3D spatial arrangement. **(C–F)** The total micro-CT 3D reconstruction illustrates the grid-like formation of new bone between the hollow scaffolds in the bone defect area from various perspectives. (Red: hollow materials. White: new bone).

Consequently, based on the pathophysiological processes of bone repair, we propose a novel and innovative modification concept for BTE scaffolds, wherein osteogenesis is based on a bone temporary shelter. This can also be referred to as the empty shell osteogenesis concept. In order to further elucidate the hollow shell osteogenesis concept proposed, we initially developed hollow spherical scaffolds designed to replicate the composition of natural bone tissue, consisting of hydroxyapatite (HA) and tricalcium phosphate (TCP) (Figures 1A, D). These scaffolds possess a rough surface structure that promotes cell adhesion (Figure 1B), a specific degradation rate (Figure 1C), and favorable biocompatibility (Figure 1E). Specifically, during the initial phase of bone regeneration, the BTE scaffold simulates autogenous bone chips, serving as a temporary supporting structure that facilitates grid-like bone reconstruction. This scaffold offers an osteoconductive platform, promoting the generation of new bone and ultimately achieving the establishment of a grid-like cancellous bone-like structure (Figures 2A, B). During the intermediate phase of bone reconstruction, the introduction of new bone into the scaffold surface pores occurs, facilitated by the hollow three-dimensional structure of the scaffold. This structure effectively directs and stimulates the inherent regenerative capacity of the bone tissue, thereby preventing the undesired occurrence of “isolated islands” (Figures 2A–F). In the late phase of bone reconstruction, under optimal circumstances, complete degradation of the hollow material occurs, thereby emulating the natural progression of autologous bone graft growth.

**FIGURE 3:**
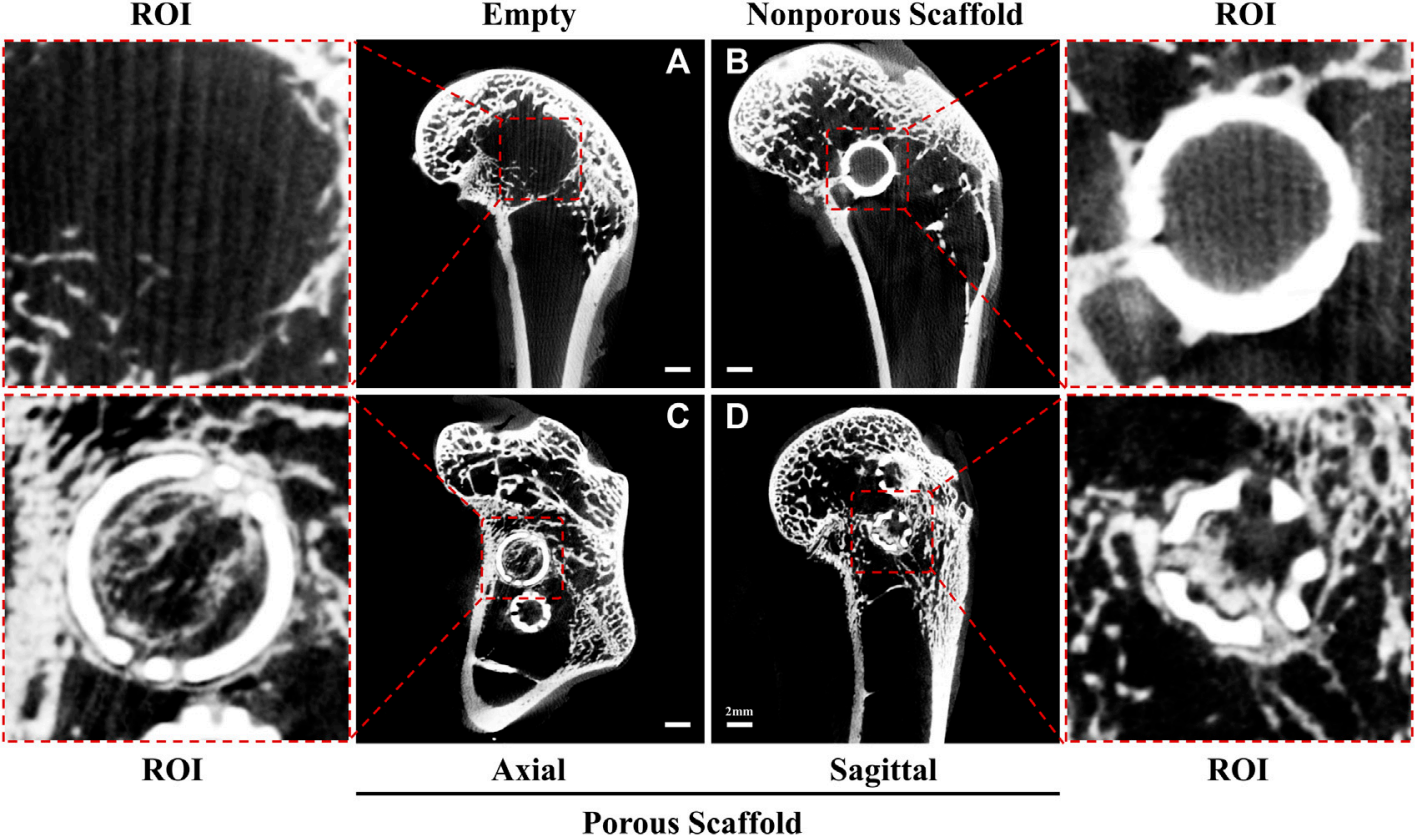
Micro-computed tomography images at 12 weeks. **(A)** Empty group: the bone defect area was unable to undergo self-repair, as evidenced by the absence of grid-like new bone formation within the ROI. **(B)** Nonporous Scaffold group: the surface of the nonporous scaffold displayed characteristics resembling a residual scaffold owing to the “isolated islands” effect. Additionally, a limited amount of new bone tissue growth was observed on the surface, while no new bone formation occurred within the scaffold itself. **(C, D)** Porous Scaffold group: a hollow scaffold featuring apertures on its surface allowed for the growth of new bone along the scaffold’s surface and inward into the new bone through the apertures. Moreover, the new bone formed a grid-like network inside the hollow structure and externally. This observation was made in the axial slice **(C)** and sagittal plane **(D)**.

To describe our hypothesis, we conducted experiments using twelve 24-week-old male New Zealand rabbits (weight: 2.5 ± 0.5 kg), and established three groups: an empty group with no intervention, a group treated with a non-porous hollow spherical HA-TCP scaffold and the other with a surface-porous hollow spherical HA-TCP scaffold. A surgical drill was used to create a cylindrical defect measuring 8 mm in diameter, ensuring that the contralateral cortex in the distal femur remained intact. Micro-CT tomography scans conducted 12 weeks post-operatively, revealed that the bone defect in the Empty group did not undergo spontaneous healing in the absence of scaffold implantation. This finding indirectly highlights the importance of employing materials that can serve as temporary osteoconductive platforms (Figure 3A). Additionally, to simulate the residual scaffolding of the “isolated island” effect, we developed a non-porous spherical scaffold. It is worth noting that we designed the scaffold as a hollow structure to facilitate observation and comparison of internal osteogenesis. These hollow scaffolds, as anticipated, led to the formation of new bone on their surface while no new bone growth occurred internally (Figure 3B). Simultaneously, we developed a spherical hollow scaffold material with porous structure. The tomographic CT scans, taken from both axial (Figure 3C) and sagittal (Figure 3D) perspectives, revealed that newly formed bone grew along the scaffold surface and established connections between them, as well as penetrated the porous structure, resulting in the establishment of a three-dimensional grid-like network within the inner region of the hollow space. This outcome closely mimics the desired process of autologous bone grafting.

Based on our theoretical considerations and preliminary experimental results, we believe that traditional BTE scaffold design strategies should be modified to fully simulate the process of autologous bone reconstruction. Therefore, we considered that the design of hollow structural scaffolds, which has been authorized by the State Intellectual Property Office of China (patent number: ZL202122966951.2), can truly prevent the “isolated island” effect. The strengths of these scaffolds are: 1. The hollow structural particles are in point contact with each other in the three-dimensional structure, which provides ample space for the tissue fluid to connect with the scaffolds and optimally mimic the extracellular matrix environment; 2. The hollow structural design of the BTE scaffolds, especially the spherical hollow design, allows for maximum contact area between tissue fluid and stem cells in the bone defect space, providing an optimal environment for cell adhesion, proliferation, and nutrient delivery; 3. The hollow spherical granular scaffolds are more suitable for repairing complex bone defects, with good mobility and easy operation; 4. The hollow structure provides a temporary location for adhesion between endothelial progenitor and bone marrow stromal cells (Qin and Zhang, 2017), and offers mechanical support comparable to that offered by bone chips; 5. As the scaffolds degrade, the new bone gradually forms a grid-like structure in the bone defect area under the guidance of the scaffold’s surface, which will not hinder the ingrowth of the new bone; and 6. By adjusting the thickness, composition, and construction of hollow scaffolds, we can match the scaffold degradation rate to the osteogenesis rate.

## 4 Conclusion

BTE scaffolds are associated with the “isolated island” effect and thus, traditional design concepts (focusing only on the material) are inadequate in facilitating bone regeneration and reconstruction. We have established the empty shell osteogenesis concept from the perspective of simulating the pathophysiological process of bone repair. In other words, osteogenesis is based on a bone temporary shelter. Consequently, we considered that designing hollow structural scaffolds is the key to mitigating the “isolated island” effect. The hollow design structure provides a basis for further development of BTE scaffolds with improved osteogenesis properties.

## Data Availability

The original contributions presented in the study are included in the article/Supplementary material, further inquiries can be directed to the corresponding authors.
